# The Conundrum of Cancer-Associated Thrombosis: Lesson Learned from Two Intriguing Cases and Literature Review

**DOI:** 10.3390/diseases12030047

**Published:** 2024-02-28

**Authors:** Simona Laurino, Sabino Russi, Ludmila Carmen Omer, Alberto D’Angelo, Giovanni Bozza, Giuseppina Gallucci, Geppino Falco, Giandomenico Roviello, Anna Maria Bochicchio

**Affiliations:** 1 Laboratory of Preclinical and Translational Research, IRCCS CROB Centro di Riferimento Oncologico della Basilicata, 85028 Rionero in Vulture, Italy; simona.laurino@crob.it; 2 Trial Office, IRCCS CROB Centro di Riferimento Oncologico della Basilicata, 85028 Rionero in Vulture, Italy; ludmila.omer@crob.it; 3 Department of Oncology, Royal United Hospital, Bath BA1 3NG, UK; alberto.dangelo@nhs.net; 4 Medical Oncology Unit, IRCCS CROB Centro di Riferimento Oncologico della Basilicata, 85028 Rionero in Vulture, Italy; giovanni.bozza@crob.it; 5 Cardiology Unit, IRCCS CROB Centro di Riferimento Oncologico della Basilicata, 85028 Rionero in Vulture, Italy; pina.gallucci@tiscali.it; 6 Department of Biology, Università degli Studi di Napoli Federico II, 80138 Naples, Italy; geppino.falco@unina.it; 7 Clinical Oncologic Unit, Careggi Hospital, University of Florence, 50121 Florence, Italy; giandomenicoroviello@hotmail.it; 8 Multispecialty Tumor Board, IRCCS CROB Centro di Riferimento Oncologico della Basilicata, 85028 Rionero in Vulture, Italy; annamaria.bochicchio@crob.it

**Keywords:** cancer patients, thrombophilic status of cancer patients, cancer-associated thrombosis (CAT), venous thromboembolism (VTE), arterial thromboembolism (ATE), mechanisms of thrombosis, reverse cardio-oncology

## Abstract

The correlation between cancer and venous thromboembolism (VTE) is solid, whereas the knowledge about cancer-related arterial thromboembolism (ATE) still needs a deeper investigation to clarify its pathogenesis. We describe two cases that represent useful hints for a comprehensive review of the thrombotic issue. A 75-year-old man with advanced rectal cancer treated with fluoropyrimidines suffered two catheter-related VTE events managed according to current guidelines. There was no indication for “extended” anticoagulant therapy for him, but during antithrombotic wash-out and fluoropyrimidines plus panitumumab regimen, he suffered a massive right coronary artery (RCA) thrombosis. Another patient with no cardiovascular (CV) risk factors and affected by advanced bladder cancer was treated with a platinum-containing regimen and suffered an acute inferior myocardial infarction 2 days after chemotherapy administration. He was successfully treated with primary Percutaneous Transluminal Coronary Angioplasty of RCA, discontinuing platinum-based therapy. Our observations raise the issue of cancer-associated thrombosis (CAT) complexity and the potential correlation between arterial and venous thrombotic events. Moreover, physicians should be aware of the thrombotic risk associated with anticancer therapies, suggesting that an appropriate prophylaxis should be considered.

## 1. Introduction

The link between cancer and venous thromboembolism (VTE) was first described in 1865 by Dr. Armand Trousseau [1]. It is well documented that cancer patients have higher incidence and recurrence rates of VTE [2]. These patients are also characterized by a greater likelihood of bleeding complications during VTE treatment, which impacts disease morbidity and mortality [3,4,5]. It is also largely documented that thrombotic risk is related to the type of primary tumor, the burden of metastatic disease, and the thrombogenic effect of oncologic treatments, with the highest 1-year VTE incidence observed in metastatic disease. Moreover, there are novel VTE risk factors, such as the mutations in JAK2 (V617F), MET, and KRAS genes in myeloproliferative and solid neoplasms, that may have a strong clinical impact [6,7,8]. On the contrary, the incidence of arterial thromboembolism (ATE) in cancer patients has only recently become a widely investigated issue, even though the association between ATE and VTE has long been hypothesized [9,10]. Indeed, the frequency and incidence of arterial events in VTE patients are known to be significantly higher than in the general population [11]. It has also been shown that unprovoked VTE may be the first sign of occult cancer and that arterial thromboembolic events can be detected about 5 months before cancer diagnosis, suggesting an early association in the natural history of neoplastic disease [12,13,14,15]. In a large cohort study, including both cancer patients and cancer-free individuals, a doubled risk of ATE, which also increases with patients’ age, has been reported [16]. Wang J et al. also highlighted that ATE event risk is higher in newly diagnosed cancer and in specific cancer types [17]. Cancer-associated thromboses (CAT) in the venous system have the clinical phenotypes of either deep venous thrombosis (DVT) or pulmonary embolism (PE); in the arterial system, acute coronary syndrome (ACS), ischemic stroke, and peripheral arterial thrombotic events are the main manifestations [18].

Here, we describe two patients experiencing both arterial and venous thrombotic events to draw attention to the complexity of CAT, whose phenotypes cannot be merely considered as independent entities in arterial and venous vessels (white thrombus and red thrombus, respectively). Indeed, cancer patients might not always be eligible for the guidelines, and thrombosis management can be exceedingly complex. 

## 2. Cases Presentation

### 2.1. Case 1

**Figure 1:**
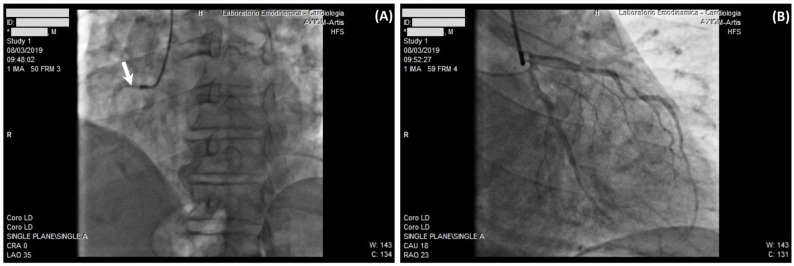
Coronary angiography showing occlusion of the right coronary artery (arrow) (**A**); normal angiogram of the left coronary artery (**B**).

A 75-year-old patient with a history of rectal bleeding was diagnosed with colon cancer in February 2018. The patient was a heavy smoker with a Body Mass Index (BMI) of 27.7 kg/m^2^ and normal values for blood pressure and cholesterol. Total-body Computed Tomography (CT) showed bilateral adrenal secondary lesions. According to the 2-month FOLFOX-4 schedule, systemic treatment was commenced in March 2018, and due to the burden of the primary tumor, although distant metastases, a chemo-radiation combined treatment was also suggested before surgery. DNA sequencing for RAS and BRAF mutations reported a wild-type pattern, thus suggesting the patient’s eligibility for the additional use of panitumumab in FOLFOX-4 therapy. A peripheral venous catheter was placed in the right basilic vein to allow the continuous infusion of fluorouracil. After a month of chemotherapy, a peri-catheter proximal thrombosis was reported. Consequently, the patient was successfully treated with 60 mg enoxaparin twice daily for 30 days, and the catheter was removed and replaced on the left side (basilic vein). Enoxaparin was continued at a prophylactic dose for 30 days. An encouraging partial response on the primary tumor, regional lymph nodes and adrenal metastases was reported following first-line chemotherapy. From 28 May 2018 to 7 July 2018, the patient received 5-fluorouracil in continuous infusion and concomitant pelvic radiotherapy in a neo-adjuvant setting. On 29 June 2018, peri-catheter proximal venous thrombosis was detected using clinical and ultrasound diagnosis, although the D-dimer tested normal. As a result, anticoagulant therapy with 60 mg of enoxaparin twice daily was administered for the following two months. The case was re-evaluated in a multidisciplinary setting, and after a three-week interruption of chemotherapy, oral fluoropyrimidine was re-started with the monitoring of venous thrombosis. Radiation treatment was regularly provided. In August 2018, an ultrasound study of the arm showed a reduction in the thrombus dimensions. The patient continued to receive a modified XELOX regimen plus enoxaparin at prophylactic dose, achieving a further shrinkage of targeted lesions. On 1 October 2018, rectal anterior resection and protective ileostomy were performed. The pathology outcome of the resected rectal segment (with regional nodes contained in the mesorectal fat) diagnosed an adenocarcinoma G3, pT3, N2, M1 (due to metastases on the adrenal glands), which required the re-start of chemotherapy with XELOX-2 plus panitumumab for an additional 3 months, followed by an abdominal CT re-staging. On 12 December 2018, due to increased pain in the left shoulder and a motor deficit in the ipsilateral upper limb, a bone scan and magnetic resonance imaging (MRI) were performed and revealed secondary bone lesions. The patient’s shoulder was treated with radiotherapy, which effectively reduced pain. Systemic treatment continued over the next months, and the CT evaluation in January 2019 showed lesion shrinkage on both adrenal glands. Consequently, the patient started maintenance treatment with capecitabine administered for 14 days combined with bi-weekly intravenous panitumumab. On 2 March 2019, for chest pain followed by syncope, the patient was admitted to the Coronary Care Unit with a final diagnosis of acute myocardial infarction (AMI) complicated by 2nd-degree atrioventricular block (type 1) and ventricular fibrillation treated with direct current (DC) shock. Coronary angiography revealed massive thrombosis of the right coronary artery (Figure 1).

Since no interventional percutaneous treatment was indicated, combined therapy with an anticoagulant (rivaroxaban) plus an antiplatelet drug (clopidogrel) was started. The investigation for thrombophilia was negative. On 10 May 2019, suspecting a relation between fluoropyrimidine administration and the AMI event, the patient received the first administration of a bi-weekly irinotecan–cetuximab regimen as a second-line treatment for advanced progressive disease. No thrombotic events were reported in the following 19 months of chemotherapy. The patient died due to the progression of the disease. 

### 2.2. Case 2

A 55-year-old patient with a healthy lifestyle, including regular agonistic physical activity and no risk factors for coronary artery disease, was diagnosed with urothelial bladder cancer (cT1G3 stage) in 2010. He was subjected to endoscopic resection and received intravesical gemcitabine as adjuvant therapy. The patient at that time refused treatment with Bacille Calmette-Guèrin due to infective risk. During follow-up in April 2018, the patient underwent Proton Emission Tomography (PET), documenting bladder uptake, and Transurethral Resection of Bladder (TURB), which revealed a low-grade bladder carcinoma. The patient was consequently treated with six administrations of Bacille Calmette-Guérin. In August 2018, due to positive urine cytology, the patient underwent a bladder cystoscopy, which documented a bulky lesion on the posterior wall of the bladder, subsequently resected using TURB. Pathologist diagnosed a sarcomatoid variant of urothelial carcinoma (pT2G3 stage), which was treated with three cycles of platinum plus a gemcitabine (CDDP/Gem) chemotherapeutic regimen (October–December 2018) in a neo-adjuvant setting followed by radical cystectomy with Orthotopic Neobladder Reconstruction (February 2019). In June 2019, a pelvic relapse was recorded and medically treated. An additional three cycles of CDDP/Gem regimen were suggested, followed by a CT re-evaluation. After two cycles of platinum, the patient complained of prolonged chest pain; an inferior ST-segment elevation myocardial infarction was diagnosed, which was successfully treated with primary Percutaneous Transluminal Coronary Angioplasty of the right coronary artery. However, platinum-based therapy was stopped due to a potential link with acute coronary syndrome, and the patient was consequently treated with Best Supportive Care. Dual antiplatelet therapy was discontinued due to thrombocytopenia, and the patient suffered from popliteal venous thrombosis. He died due to progression of the disease.

## 3. Discussion

Both patients suffered venous and arterial thrombotic events. In a preventive setting, recommendations for the venous outpatient thromboprophylaxis of cancer patients are provided by the Khorana score [19]. The 2019 ASCO guidelines (strong, evidence-based recommendations of intermediate-to-high evidence quality) state as follows: “*Routine pharmacologic thromboprophylaxis should not be offered to all outpatients with cancer and high-risk outpatients with cancer (Khorana score of 2 or higher prior to starting a new systemic chemotherapy regimen) may be offered thromboprophylaxis with apixaban, rivaroxaban, or low-molecular-weight heparin (LMWH) provided there are no significant risk factors for bleeding and no drug interactions*” [20]. The NCCN 2021 Practice Guidelines in Oncology state as follows: “*No routine VTE prophylaxis in low-risk patients* (*Khorana score* < *2*)” [21]. The first patient—Khorana score of 0—did not undergo antithrombotic prophylaxis before chemotherapy. In 2021, the ASH guidelines specifically addressed the issue of central venous catheters (CVCs), suggesting “*not using parenteral/oral thromboprophylaxis*” [22]. The first venous thrombotic event (at the proximal right basilic vein) was considered a catheter-related occurrence, being central venous access devices (CAVDs) considered a treatment-related risk factor for upper extremity CAT. Consequently, the patient received 30 days of therapeutic LMWH; then, the catheter was removed due to the complete resolution of thrombosis. A prophylactic dose of LMWH was administered for 30 days, according to the 2020 ASCO guidelines: *“Initial anticoagulation may involve LMWH, UFH, fondaparinux or rivaroxaban”* [20]. The suggested short-term duration is 3 to 6 months. Given the causal relation with the peripheral catheter, the superficial location of thrombosis (CVC-related superficial thrombosis), and a high likelihood of chemotherapy-induced thrombocytopenia, longer anticoagulant therapy was deemed not necessary. Actually, it could have been wiser to keep the catheter and prolong the administration of LMWH. In fact, catheter removal is not suggested in the 2021 ASH guidelines (conditional recommendation: “*For patients with cancer with CVC-related VTE receiving anticoagulant treatment, the ASH guideline panel suggests keeping the CVC over removing the CVC*”) [22]. As a matter of fact, the 2022 guidelines also recommend the treatment of established catheter-related thromboses for a minimum of 3 months and *as long as the central venous catheter is in place* [23]; in this setting, LMWH is suggested. The last ESMO guidelines do not recommend routine pharmacological prophylaxis of catheter-related thrombosis (CRT) [II, D], recommend anticoagulant treatment of symptomatic CRT for a minimum of 3 months [III, A], still suggesting LMWH as the first choice of therapy; the removal of catheter is indicated, if not needed or infected, in case of contraindications of anticoagulants or thrombus extension despite treatment [III, B]. These guidelines suggest to extending after 3 months the anticoagulant treatment in case of low bleeding risk [IV, C] [7]. The second event (left proximal superficial basilic vein) was also speculated to be an indwelling catheter-related occurrence; a short-term treatment of anticoagulant therapy for 3 months was administered. The patient was considered eligible neither for a long-term anticoagulant treatment nor for antithrombotic prophylaxis (Khorana score = 0). When the acute coronary syndrome was diagnosed, the patient was not under antithrombotic treatment. The occurrence of an arterial thrombotic event after two episodes of venous thrombotic events is the intriguing feature of this case and raises the issue of thrombogenicity of cancer itself. Risk scores for arterial thrombo-prophylaxis in cancer patients have not been recommended yet, but the emerging role of shared risk factors between cancer and cardiovascular disease (CVD) may pave the way for the discovery of common risk scores and of new pathways that might be therapeutically targeted. 

In the second patient, who was free of common coronary artery disease risk factors, no known features of thrombophilia, and no echocardiographic findings, we speculated that the platinum-based therapy combined with the cancer-associated thrombogenicity promoted the cardioembolic ACS (normal dimensions of cardiac chambers at different echocardiographic examinations, normal valve function, and normal aspect of the interatrial septum) [24,25]. 

Cancer itself may indeed promote arterial thrombosis. Even though cancer has not been considered an independent risk factor for ATE, recent data support the concept of a common etiopathogenetic process in cancer and atherosclerotic CVD, and the top player of the process is chronic inflammation [26,27]. Cancer patients are, indeed, more prone to CVD compared with patients without cancer, and patients with atherosclerotic CVD have an increased incidence of cancer. Navi et al. studied 279,719 patients with a recent diagnosis of cancer and compared them to a control group [28]; they found a 6-month ATE cumulative incidence of 4.7% in cancer patients versus 2.2% in the control group. The incidence of myocardial infarction was 2.0% in cancer patients while 0.7% in the control group, and ischemic stroke was 3% in cancer patients and 1.6% in the control group. The analysis reported a potential increase in myocardial infarction and ischemic stroke risk in the 6 months after cancer diagnosis and a normal range of risk after 1 year. Additionally, a different thrombosis predisposition across different types of cancers was documented, thus implying a direct effect of cancer itself on thrombotic events, along with its ability to secrete pro-coagulant factors [29]. In another study, Grilz et al. found a significantly higher Relative Risk (RR) of both ATE and VTE in patients with cancer in a nationwide analysis: RR of 6.88 for ATE and RR of 14.91 for VTE [30]. The increased thromboembolic risk in cancer patients is a complex and multifactorial phenomenon, including the release of several pro-coagulant factors by the tumor. In this scenario, prophylaxis should maximize the benefit/adverse effect ratio. Indeed, aspirin is no longer used in primary prevention due to its bleeding risk [31], whereas there is growing evidence of the use of statins for the prevention of arterial thrombosis events [32]. According to data from Brenner et al. on cancer patients with acute VTE from the Registro Informatizado de Enfermedad Trombo Embolica (RIETE), ATE is less frequent than VTE recurrence but results in a worse prognosis. It is characterized by a thirty-day mortality rate of 20% after pulmonary embolism recurrence, 40% after myocardial infarction, 64% after ischemic stroke, and 83% after lower limb amputation [33]. However, the reverse is also true: CVD-induced acceleration of cancer pathogenesis, the so-called reverse cardio-oncology [34]. This new concept has stimulated many studies to better understand the link between cancer and CVD. Besides the multiple shared risk factors (age, smoking, diabetes, hypertension, hypercholesterolemia, obesity, physical inactivity, unhealthy diets, alcohol abuse, impaired immune response, metabolic remodeling, and clonal hematopoiesis), there is burgeoning research on the shared molecular mechanisms of the two diseases. Narayan et coll. have defined the following shared biological processes between cancer and CVD [35]: inflammation, cellular proliferation, and resistance to cell death, with the Growth Differentiation Factor-15 (GDF-15) as a peculiar marker with a prognostic role in cancer and in cardiovascular diseases [36], neurohormonal stress, angiogenesis, and genomic instability. The increased thromboembolic risk in cancer patients is a complex and multifactorial phenomenon, including the release of several pro-coagulant factors by tumors [29]. Since ATE risk is higher in the early months after cancer diagnosis, psychological stress affecting cancer patients can represent a trigger for ATE events [37]. Thrombus formation is the final stage of complex interactions between coagulation, fibrinolysis, and innate immune systems. In this intriguing scenario, leukocyte recruitment is a key step; recent studies have conferred a strategic role to neutrophils in both venous and arterial CAT. Neutrophils role in immune-thrombosis is mediated by the release of neutrophil extracellular traps (NETs), a net-like structure consisting of DNA filaments including superimposed histones and granule proteins [38,39,40,41]. NETs provide a scaffold for platelets, red blood cells, and pro-coagulant molecules [42,43,44,45]. Moreover, tumor-induced NETosis might have a relevant role in the process of metastasis spreading [46]. Other important players in thrombosis are Extracellular Vesicles (EVs), which have been described as crucial mediators of intercellular communication and modulators of inflammation, vascular dysfunction, and thrombosis [47]. Recent studies have documented that EVs released by cancer cells could promote a pro-coagulant shift on endothelium, which expresses Tissue Factor (TF) and thrombin. Moreover, it has been demonstrated that cancer cells release TF-enriched EVs, which activate platelets and favor their aggregation [48]. Lastly, we must report that the thrombotic risk of chemotherapy and other cancer treatments has still to be addressed. Tamoxifen increases VTE risk by two- to seven-fold, with an uncertain impact on arterial thrombosis. On the other hand, immunomodulatory imide drugs such as thalidomide and lenalidomide increase the risk of VTE in patients diagnosed with multiple myeloma when administered in combination with glucocorticoids or other chemotherapy agents. Regarding panitumumab, there is a growing concern about a possible correlation between this agent and an increased risk of venous thrombosis. Other drugs with vasculo-toxic potential include antiangiogenic and erythropoiesis-stimulating agents, granulocyte colony-stimulating factors, and steroids [49].

Immune checkpoint inhibitors (ICIs) have unveiled some features of the complex relationship between the immune system, inflammation, and atherosclerosis. Preclinical studies have shown that the targets of ICIs (CTLA-4, PD-1, LAG-3, and PD-L1) are proteins with a negative regulatory role in atherosclerosis [50]. The blockage of the checkpoints may predictably cause accelerated atherosclerosis due to enhanced T cell responses, limited Treg function, and the infiltration of the vascular endothelium [51,52,53,54]. This effect can be considered a phenotype of arterial damage due to ICIs. As far as the VTE risk in ICI treatment is concerned, a recent retrospective cohort study using HealthCore Integrated Research Environment—Oncology data from 2299 eligible patients with first-line treatment of stage IV non-small cell lung cancer found that the risk of VTE was 26% lower for ICI-based regimens when compared to chemo-based regimens with or without ICIs [55]. However, in contrast, in other real-life cohorts, substantial rates of VTE and ATE under ICI therapy emerged [56]. Moreover, the advent of targeted anticancer regimens is defining a new patient population at higher risk of cancer-associated thrombosis, both VTE and ATE. Indeed, as recently reviewed, increased rates of thromboembolic events have been described for several targeted and immunotherapeutic regimens [56,57]. 

## 4. Conclusions

CAT is a complex and multifactorial process that increases morbidity and mortality, leads to the interruption of cancer treatment, decreases quality of life, and raises healthcare costs. Therefore, it is crucial to increase our awareness of VTE and ATE. For VTE prediction, it is important to improve the Risk Assessment Methods (RAMs) by including old and novel biomarkers to better identify cancer outpatients with a high risk of VTE and taking into account cancer itself and cancer treatment-associated thrombotic risk [6]. For ATE prediction, we do not have scores yet, but it is mandatory to assess the “arterial thrombotic risk” in cancer patients at baseline, with careful identification of the common risk factors [58]. Additionally, the intriguing relationship between cancer and CVD, the shared risk factors, and the underlying molecular mechanisms that the recent studies on ICIs have shed light on will pave the way for new markers and new treatments. Cancer patients and CAT management can be exceedingly challenging; a multidisciplinary approach may help to find the best-tailored weaponry both for prophylaxis and treatment. This strategy should include the aggressive treatment of all CV risk factors and judicious and guideline-driven use of direct oral anticoagulants and antiplatelet agents, taking into account drug interferences and suboptimal absorption. The goal is to reduce the burden of CATs and its crucial impact on cancer patients.

## Data Availability

All data generated or analyzed during this study are included in this published article.
